# Structural Analysis of Breast-Milk α_S1_-Casein: An α-Helical Conformation Is Required for TLR4-Stimulation

**DOI:** 10.3390/ijms25031743

**Published:** 2024-02-01

**Authors:** Thorsten Saenger, Marten F. Schulte, Stefan Vordenbäumen, Fabian C. Hermann, Juliana Bertelsbeck, Kathrin Meier, Ellen Bleck, Matthias Schneider, Joachim Jose

**Affiliations:** 1 Institute for Pharmaceutical and Medicinal Chemistry, University of Münster, PharmaCampus, Correnstr. 48, 48149 Münster, Germany; saenger.thorsten@mh-hannover.de (T.S.); marten.schulte@gmx.de (M.F.S.);; 2 Department of Rheumatology and Hiller Research Unit Rheumatology, Medical Faculty, Heinrich-Heine-University Düsseldorf, Moorenstr. 5, 40225 Düsseldorf, Germany; 3 Institute for Pharmaceutical Biology and Phytochemie, University of Münster, PharmaCampus, Correnstr. 48, 48149 Münster, Germany

**Keywords:** breast milk, α_S1_-casein, structure analysis, α-helical content, TLR4

## Abstract

Breast-milk α_S1_-casein is a Toll-like receptor 4 (TLR4) agonist, whereas phosphorylated α_S1_-casein does not bind TLR4. The objective of this study was to analyse the structural requirements for these effects. In silico analysis of α_S1_-casein indicated high α-helical content with coiled-coil characteristics. This was confirmed by CD-spectroscopy, showing the α-helical conformation to be stable between pH 2 and 7.4. After in vitro phosphorylation, the α-helical content was significantly reduced, similar to what it was after incubation at 80 °C. This conformation showed no in vitro induction of IL-8 secretion via TLR4. A synthetic peptide corresponding to V^77^-E^92^ of α_S1_-casein induced an IL-8 secretion of 0.95 ng/mL via TLR4. Our results indicate that α_S1_-casein appears in two distinct conformations, an α-helical TLR4-agonistic and a less α-helical TLR4 non-agonistic conformation induced by phosphorylation. This is to indicate that the immunomodulatory role of α_S1_-casein, as described before, could be regulated by conformational changes induced by phosphorylation.

## 1. Introduction

Human α_S1_-casein is a breast-milk protein, which was described to be overexpressed in the synovia of patients with osteoarthritis or rheumatoid arthritis [1,2], the blood of patients with multiple sclerosis [3], in the tissue of patients with benign prostate hyperplasia [4] or breast cancer [5]. Furthermore, α_S1_-casein was reported to have an immunostimulatory role, as it appeared to induce a lifelong human immunoglobulin G (IgG)-antibody response due to breastfeeding [6] and secretion of several cytokines via Toll-like receptor 4 (TLR 4), e.g., granulocyte macrophage colony-stimulating factor, interleukin 1β, interleukin 6 and chemokine IL-8 (interleukin 8) [7,8]. The α_S1_-casein-induced cytokine secretion via TLR4 was blocked by phosphorylation with human protein kinase CK2 [8]. Therefore, it was postulated that α_S1_-casein could have two functions, a TLR4-agonistic, most likely immune modulatory function when unphosphorylated and a mere nutritional function when phosphorylated [9].

Human α_S1_-casein and its primary structure were discovered lately after other caseins in breast milk [10,11]. The studies focused on the quantification of α_S1_-casein in breast milk, its posttranscriptional [11,12] and posttranslational modification. Hints for oligomers and disulfide bonds were mentioned. Furthermore, the authors described α_S1_-casein in the matrix of breast milk as more stable than other breast-milk proteins, in particular with respect to acidic pH, breast-milk proteases and Holder pasteurization [11,12,13,14,15]. A higher state of organization, however, in the structure of, e.g., purified human α_S1_-casein was not investigated. A reason for this could be the low content of breast-milk α_S1_-casein with less than 1% (3 mg/L to 537 mg/L) of total milk protein content [16].

Bovine α_S1_-casein was intensively studied with regard to various features, including casein micelle formation, secondary structure, interaction with itself, phosphorylation, chaperone activity, intrinsically disordered regions, and fibrillation [14,17,18,19,20,21,22,23,24]. This was reasoned in its easy accessibility and due to its prominent role as food (e.g., infant formula) as well as a major food allergen [19,20,25,26]. In contrast to human α_S1_-casein, bovine α_S1_-casein is a major protein of bovine milk with around 30% of total milk protein content [16]. The AAS of bovine and human α_S1_-casein share less than 30% homology [27]. The missing cross-reactivity of IgG to human α_S1_-casein in human sera and IgG to bovine α_S1_-casein [27] showed that these two proteins do not share IgG-epitopes. Hence, they had different exposed secondary, tertiary or quaternary structural motifs.

The aim of this study was the characterization of human α_S1_-casein structure in relation to its two functions, immune active (TLR4-agonistic) and nutritional (nonagonistic). The question to clarify was, can the two functions of α_S1_-casein be related to two distinct conformations and is the switch between the two conformations eventually due to phosphorylation. To some extent, the structure of bovine and human α_S1_-casein was compared to gain further insights into α_S1_-casein species specificity. This could help to understand the role of human α_S1_-casein, its phosphorylation-dependent modulation and regulation of immune activity in early infancy and pathogenesis. Moreover, it would give a prime example of how two completely different functions of a single protein are regulated by phosphorylation or dephosphorylation, respectively.

## 2. Results and Discussion

### 2.1. In Silico and Experimental Structure Analysis of Human α_S1_-Casein

#### 2.1.1. Amino Acid Sequence Analysis and Phylogenetic Relationships

**Figure 1:**
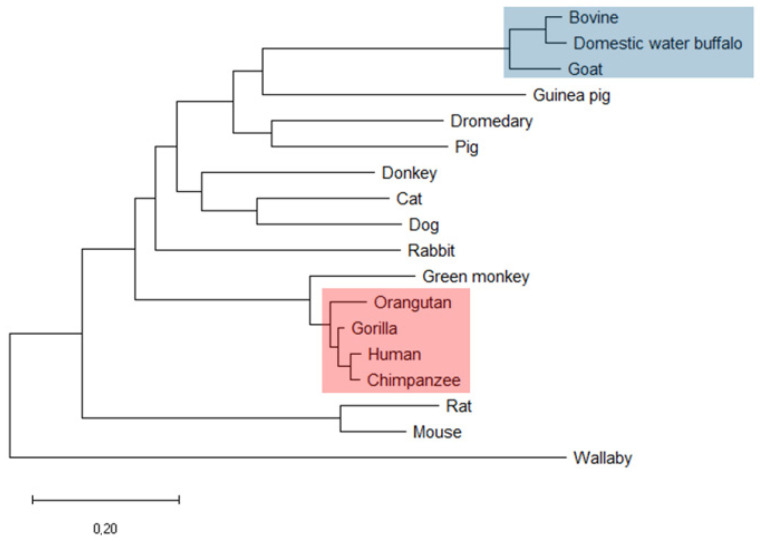
Phylogenetic tree as obtained by comparison of human α_S1_-casein amino acid sequence with that from 17 other species (*red:* hominids; *blue:* bovidae). It was calculated using molecular evolutionary genetic analysis.

First, the amino acid sequence (AAS) of human α_S1_-casein was compared to the AAS of α_S1_-caseins from 17 other species using molecular evolutionary genetics analysis software [28], with results shown in Figure 1 and Figure S1. Overall, the phylogeny of α_S1_-casein was in accordance with the phylogenetic tree obtained by analysing 1% of the human genome [29]. Exceptions were mouse and rat α_S1_-casein, which showed only low AAS homology to rabbit α_S1_-casein. AAS of human α_S1_-casein had an identity of more than 92% to AAS of non-human hominids such as α_S1_-caseins from chimpanzee (97%), western lowland gorilla C95%), and Sumatran orangutan (92%). AAS of primate *Chlorocebus sabaeus* (green monkey) had an identity of 82% to human α_S1_-casein but exhibited no probability for a coiled-coil region. All other species had an identity below 50% to human α_S1_-casein, e.g., rabbit (42%), mouse (29%) and rat (27%), donkey and dromedary (39%), cow (33%) and pig (35%). Furthermore, a coiled-coil domain was predicted for α_S1_-casein in the same AAS region in all four hominids investigated (Figure S1). A coiled-coil region was also predicted for rabbit, guinea pig and donkey α_S1_-casein. But here, the coiled-coil was predicted at a different position in the AAS compared to human α_S1_-casein. In conclusion, high identity in the AAS and secondary structure elements as a coiled-coil in hominids could be a hint that these α_S1_-casein share structure and, consequently, function which could be different from other mammals.

#### 2.1.2. Hydrophobicity Analysis of Human α_S1_-Casein and Comparison with Bovine α_S1_-Casein

**Figure 2:**
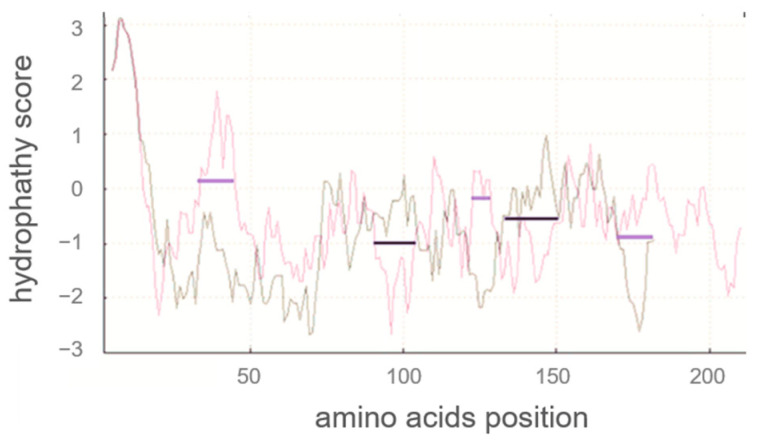
Comparison of the hydrophilicity plots of human (grey) and bovine α_S1_-casein (red) with signal peptide using Kyte Doolittle scale (hydrophobic > 0; hydrophilic < 0; window of nine amino acids; Expasy (SIB, Lausanne, Switzerland)). Black lines showing positions where human α_S1_-casein showed higher hydrophobicity and violet lines showing positions where bovine α_S1_-casein showed higher hydrophobicity.

Next, a hydrophobicity plot of human α_S1_-casein was calculated Expasy (SIB, Laussane, Switzerland) with Kyte and Doolittle coefficients [30], as illustrated in Figure 2 and compared to bovine α_S1_-casein. This hydrophobicity plot could give an indication of (1) which AA of human α_S1_-casein could be exposed, (2) whether S^33^, S^41^, S^71^ and S^89^ could be accessible for phosphorylation, and (3) which parts of α_S1_-casein were more hydrophobic and could be involved in intra- and/or intermolecular binding (e.g., to other human caseins). Human α_S1_-casein contained more hydrophilic AA at the N-terminus, especially at AAS 30–45, and the C-terminus (at AAS 170–185) compared to bovine α_S1_-casein. Human α_S1_-casein was more hydrophobic at positions 83–102 compared to bovine α_S1_-casein. This was the region a helix–loop–helix motive (SSS-X-EE) reported to be highly conserved in α_S1_-casein [11]. Although this region is highly conserved, the neighbouring AA of this motive shows weak similarity between human and bovine α_S1_-casein. AAS 30–45 containing two phosphorylation sites (S^33^ and S^41^) could be exposed more to hydrophilic solutions compared to S^89^. Therefore, S^33^ and S^41^ could be more easily accessible for kinases. In accordance, it was found before that S^33^ and S^41^ of human α_S1_-casein [31] and stretch 85–102 of bovine α_S1_-casein were phosphorylated in significant amounts. Opposite to this, only low levels of phosphorylation were described for the hydrophobic stretch 83–102 of human α_S1_-casein, especially for S^89^ [14,31]. The low level of phosphorylation in stretch 83–102 of human α_S1_-casein indicated that it was less accessible for kinases, although it was found to be more hydrophobic than in bovine α_S1_-casein. Less accessibility of kinases could be caused by intra- or intermolecular binding of human α_S1_-casein stretch 83–102. In total, human α_S1_-casein contained more hydrophilic AA compared to bovine α_S1_-casein. The differences in hydrophobicity and the phosphorylation of human and bovine α_S1_-casein, as reported, suggested that they interact with other proteins or with themselves in a different manner.

#### 2.1.3. In Silico Secondary Structure Analysis of Human α_S1_-Casein

**Figure 3:**
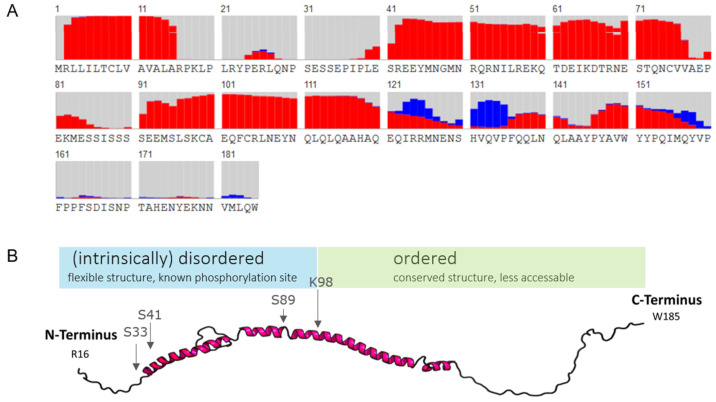
Analysis of the secondary structure. (**A**) Secondary structure prediction of the AAS of human α_S1_-casein with signal peptide (grey: random coil structure; red: α-helix; blue: β-sheet). (**B**) Prediction of tertiary structure of human α_S1_-casein using RaptorX.

Finally, secondary structures of human α_S1_-casein were predicted by RaptorX protein simulation [32,33], as shown in Figure 3. The probability for α-helical content of α_S1_-casein was highest at AAS S^41^-V^77^ and AAS S^90^-E^121^, as well as the probability for random coils at AAS R^16^-E^40^, A^78^-S^89^, and V^158^-W^185^. Moreover, the probability for a β-sheet content was predicted at AAS I^123^-P^135^. In comparison to bovine α_S1_-casein (Figure S2), human α_S1_-casein appeared to have a higher α-helical content and longer α-helical stretches. For illustration, the first model of human α_S1_-casein based was constructed, using the PDB (protein data bank)-based web service RaptorX (Figure 3B). The model was in accordance with the secondary structure predictions, supporting a high α-helical and a high random coil content in α_S1_-casein. A *p*-value below 0.001 is an indicator that the model is accurate in describing an α-helical protein [32,33]. However, the accuracy of the secondary structure prediction was low in our case because the template as used (PDB: 5DFZ) had low similarity to human α_S1_-casein, with a *p*-value of 0.019. Until now, the tertiary structure of α_S1_-casein is unknown and lacks any better similarity to a known structure of the PDB or other structures described in the dark proteome databank [34].

#### 2.1.4. Prediction and Probability for Intrinsically Disordered Regions or Transmembrane Domains

**Figure 4:**
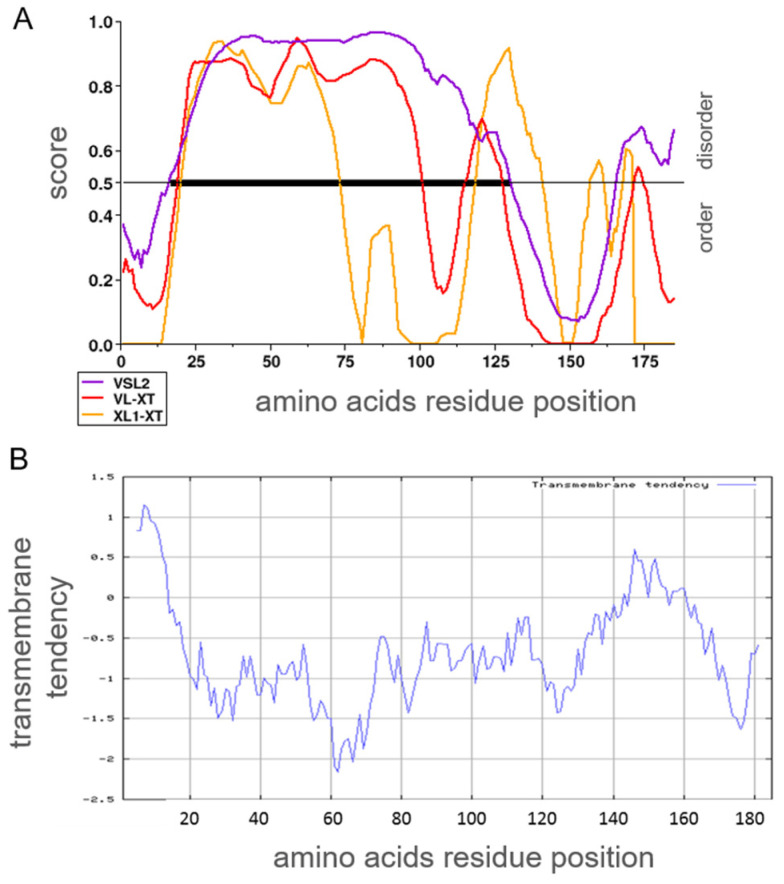
(**A**) Prediction of human α_S1_-casein for its probability as intrinsically disordered using different training sets of PONDR (yellow: algorithm XL1 optimized to predict domains longer than 39 amino acids; red: algorithm VLXT valid for proteins being completely disordered; purple: Algorithms VSL2 combining both algorithms). Black line: region with high probability to be intrinsically disordered. (**B**) Probability of human α_S1_-casein to form transmembrane regions.

A lack of similarity to any known structure can be due to an incomplete characterization of a protein. Nonpredicted transmembrane domains or intrinsically disordered regions could mislead structural comparison. Hence, the AAS of human α_S1_-casein was analyzed for the probability of acquiring an intrinsically disordered conformation (Figure 4A) and whether such a region of disorder could be set into relation of a possible phosphorylation site using three algorithms of PONDR [35] (Molecular Kinetics Inc., Indianapolis, IN, USA). With high probability, human α_S1_-casein was predicted to be an intrinsically disordered protein. All phosphorylation sites of human α_S1_-casein known so far were located within AAS 16–125 with a high probability of being intrinsically disordered. A transmembrane domain could be excluded when the AAS of human α_S1_-casein was analyzed with the algorithm of EXPASY (SIB, Lausanne, Switzerland), described by Zhao and London [36], as shown in Figure 4B.

It can be assumed that α_S1_-casein present in colostrum breast milk is unphosphorylated because, in a former analysis, high ratios of peptides with phosphorylation sites were detected in their unphosphorylated form [31].

#### 2.1.5. Secondary Structure Analysis of Human α_S1_-Casein by Spectroscopic Methods

**Figure 5:**
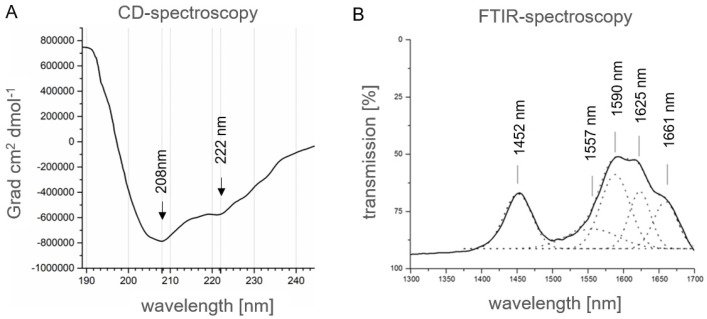
Spectroscopic analysis of human α_S1_-casein. (**A**) CD-spectra of recombinant human α_S1_-casein (12.5 µM; 0.2 mm path length). (**B**) ATR-FTIR-spectra of human α_S1_-casein (200 µM). By deconvolution, the typical peaks of the amide-I band were obtained (dotted line), which showed maxima at 1452 nm, 1557 nm, 1590 nm, 1625 nm and 1661 nm.

Therefore, recombinant α_S1_-casein from *E. coli*, which is a priori not phosphorylated, was used for experimental verification of the structure obtained in silico, as described above. For this purpose, the secondary structure of recombinant human α_S1_-casein (12.5 µM in 10 mM NaH_2_PO_4_/Na_2_HPO_4_, pH: 7.4, 20 °C) produced in *E. coli* was analyzed by CD-spectroscopy. As shown in Figure 5A, the spectrum as obtained showed typical minima for high α-helical content of a protein at 207 nm (−729,060 Grad·cm^2^·dmol^−1^) and 222 nm (−489,105 Grad·cm^2^·dmol^−1^) with a ratio (222 nm/208 nm) of 0.74 (Figure 5A). To estimate secondary structural features, the CD spectra obtained were compared to the protein data bank using K2D3 [37]. The CD-spectrum of α_S1_-casein showed a high proportion of 91.3% of α-helical content. Minima at 207 nm showed a more than 8 times more intense molar ellipticity compared to predicted spectra. Such high molar ellipticity suggests specific molecular interactions, which could be reasoned in intra- or intermolecular helix–helix interaction [38]. Overall, CD-spectra of human α_S1_-casein indicated a higher α-helical content in comparison to recombinant bovine α_S1_-casein, which was reported before to have only a minimum at 205 nm [23], typical for proteins with low or no α-helical content.

In consequence, a further secondary analysis of human α_S1_-casein was performed by ATR-FTIR (Attenuated Total Reflection-Fourier Transformation Infra-Red spectroscopy, Figure 5B) because it is known that CD-spectroscopy tends to overrate α-helical content and to underestimate β-sheet content of proteins. In the spectra as obtained, the resolution of the amide band I (1600 to 1700 cm^−1^) showed a maximum at 1661 cm^−1^, which was a strong indication for a 3_10_ α-helical structure. Nevertheless, a maximum at 1625 cm^−1^ indicated that α_S1_-casein was at least partially β-sheet structured. To sum up, in silico structure prediction, CD and ATR-FTIR spectra concordantly indicated a high α-helical and partially random coil content of human α_S1_-casein, and in addition, that it is partially β-sheet structured. The in silico structure analysis suggested that α_S1_-casein is an intrinsically disordered protein. Moreover, the secondary structure, as well as degree and pattern of phosphorylation of human α_S1_-casein was different from bovine α_S1_-casein hypothetically, resulting in a difference in function.

### 2.2. Oligomerization of Human α_S1_-Casein

Heteromers of human α_S1_-casein, e.g., with κ-casein [14] or milk micelles containing α_S1_-casein and other caseins [39] have been reported. In order to find out, at first, whether the high molar ellipticity would be a result of intermolecular interaction of α_S1_-casein with itself, we tried to substantiate oligomers of α_S1_-casein by SDS-PAGE (sodium dodecyl sulfate–polyacrylamide gel electrophoresis), MST (microscale thermophoresis) and SPR (surface plasmon resonance spectroscopy).

**Figure 6:**
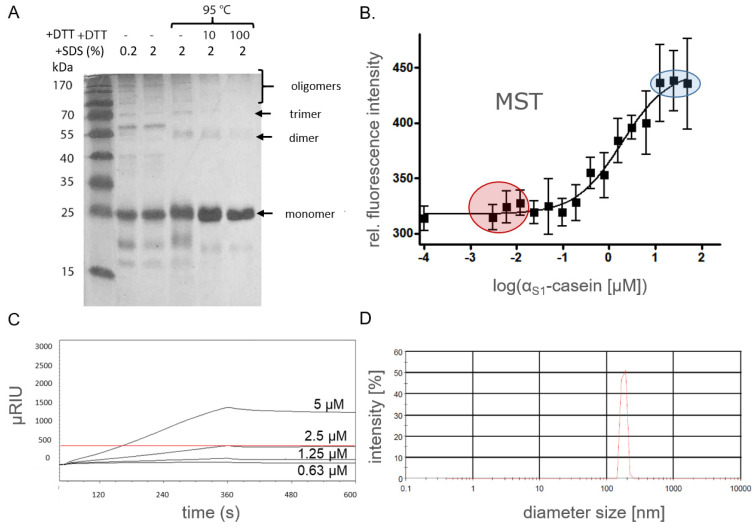
Oligomerization of human α_S1_-casein. (**A**) α_S1_-casein (50 µM) was denaturized using 0.2% SDS (20 min, RT), 2% SDS (20 min, RT) and 2% SDS (0 to 100 mM DTT, 20 min, 95 °C). These samples were analyzed by silver-stained SDS-PAGE (15%). (**B**) FITC-α_S1_-casein (25 nM) was added to α_S1_-casein (50 nM to 50 µM), sonicated for 15 min, incubated for 1 h at 37 °C and analyzed by MST (red: plateau of α_S1_-casein monomer; blue: plateau of α_S1_-casein oligomer formation). (**C**) α_S1_-Casein (450 µRIU) was immobilized on a SPR sensor chip. Concentrations of α_S1_-casein (0.63 to 5 µM) were injected. Association (5 min, 5 µL/min) and dissociation (5 min, 5 µL/min) were monitored by SPR (red line: plateau of α_S1_-casein dimer formation). (**D**) Oligomer diameter of α_S1_-casein (50 µM, pH 7.4, 37 °C) was analyzed by PCS.

For oligomer analysis by SDS-PAGE, human α_S1_-casein (50 µM) was diluted in sample buffers under different denaturing conditions (Figure 6A): 0.2% SDS (20 min, RT), 2% SDS [20 min, RT] and 2% SDS (100 mM dithiothreitol, DTT, 20 min, 95 °C). After incubation at low SDS content and lower temperatures SDS-PAGE of α_S1_-casein led to prominent bands at molecular weights of 25, 55, 70 kDa, as well as several bands at even higher molecular weights. These bands were assigned to α_S1_-casein monomers, dimers, trimers and oligomers of higher order. The intensity of bands assigned to oligomers disappeared with stronger denaturation conditions, whereas the band assigned to the monomer appeared with stronger intensities. Only a single band at 25 kDa remained after treatment with sample buffer representing the strongest denaturation conditions (2% SDS, 100 mM DTT, 20 min, 95 °C). This was a clear indication that the bands detected at higher molecular weights under weaker denaturation conditions were indeed α_S1_-casein oligomers. The intensity of the monomer band was lower, the weaker the denaturation conditions were as applied, whereas bands assigned to oligomers appeared with higher intensities. This seemed to be a clear indication for oligomerization of αS1-casein under nondenaturing and probably as well under native conditions [40]. Therefore, α_S1_-casein can be considered to form oligomers. The required addition of a reducing reagent as DDT for complete α_S1_-casein monomerization could be an indication of disulfide bonds between different α_S1_-casein monomers. Disulfide bonding has been described before for bovine α_S2_-casein [41] but not for bovine α_S1_-casein due to the overall lack of cysteines in this casein.

Next, FITC (fluorescein isothiocyanate)-labeled α_S1_-casein (25 nM) was added to different concentrations of nonlabeled α_S1_-casein (50 nM–175 µM), sonicated for 15 min, incubated for 1 h at 37 °C and analyzed by MST (Figure 6B). The binding of nonlabeled α_S1_-casein to the fluorescence-labeled isoform was clearly detectable by this method, and a K_D_ value of 2.2 µM was determined. As a second method for supporting the binding of α_S1_-casein to itself, SPR was applied (Figure 6C). α_S1_-casein was immobilized on a CMDP-5 sensor chip, which could be monitored by a signal increase of 450 µRU. Subsequently, different concentrations of unlabeled α_S1_-casein were injected (0.63 to 5 µM). By this method, a K_D_ value of 2 µM was determined. This was in good agreement with the K_D_ value of 2.2 µM as determined by MST. As the signal increase is directly proportional to the mass, one would expect a signal increase of 450 µRU for an α_S1_-casein dimer. The signal increased up to 1300 µRU and was, therefore, three times as high as with immobilized α_S1_-casein. Therefore, SPR hints at the formation of tetramers. Due to the several types of α_S1_-casein oligomers as identified by SDS-PAGE and by SPR, one has to consider that the K_D_ value 2 µM could be due to a mixture of dimer, tetramer and higher oligomer. K_D_ value was not obtained for dimer formation. The K_D_ value of unphosphorylated α_S1_-casein binding to itself was in the same order of magnitude as the K_D_ value of 2 µM for dephosphorylated bovine α_S1_-casein determined by SPR [21] and postulated K_D_ values for all bovine caseins between 1 and 3 µM [21,42].

We observed that injection of α_S1_-casein, which was stored longer than 12 h at 4 °C resulted in a loss of signal in the SPR sensograms and clogging of injection needles and capillaries. The formation of larger α_S1_-casein oligomers could have been a reason for this. It would be in accordance with the results of the SDS-PAGE analysis, where higher α_S1_-casein oligomers (e.g., octamers) were detected. Therefore, α_S1_-casein oligomers were investigated for their diameter by PCS (photon correlation spectroscopy, Figure 6D). Using this method, α_S1_-casein oligomers were detectable with a mean diameter (Ø) of 73.4 nm (polydispersion index [PI]: 0.6).

In summary, recombinant α_S1_-casein, when unphosphorylated, formed higher-order oligomers with a moderate affinity of α_S1_-casein to itself (K_D_ value of 2 µM). The α_S1_-casein oligomers had a considerable diameter of 73.4 nm. This could indicate that α_S1_-casein functions could be impaired by its affinity to itself (protein–protein interaction) and/or influence the formation and diameter of micelles, e.g., in breast milk [39].

### 2.3. The Influence of Temperature, pH and Phosphorylation on α_S1_-Casein Structure

**Figure 7:**
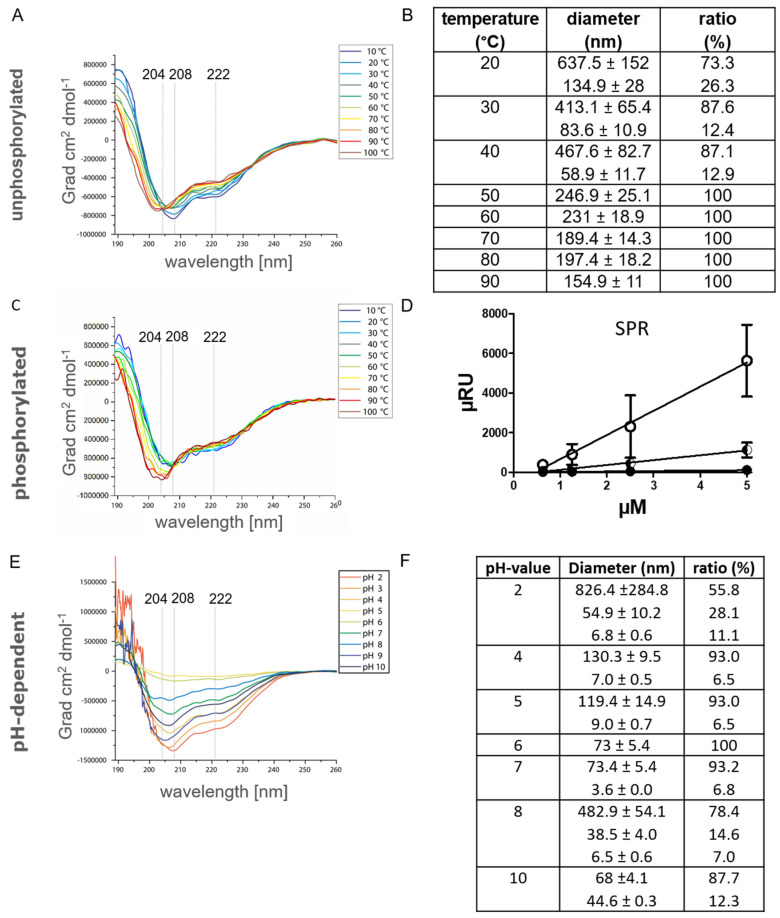
Structural characteristics of human α_S1_-casein in dependence of temperature (**A**,**B**), phosphorylation (**C**,**D**) and pH value (**E**,**F**). A, C, E: CD-spectra of human α_S1_-casein (12.5 µM; 2 mm path length). B, F: Diameter detected of 50 µM α_S1_-casein by PCS at pH 7.4 and different temperatures (**B**) as well as at different pH at 37 °C (**F**). D: α_S1_-casein was immobilized on a SPR sensor chip and concentrations from 0.625 µM to 5 µM of α_S1_-casein were injected. Dots represent the difference in the signal intensity at steady-state (binding) compared to coated chip ([•] α_S1_-casein binding itself; [○] P-α_S1_-casein binding itself).

In previous works, it was shown that α_S1_-casein binding to TLR4 was abrogated by heat denaturation [8]. Therefore, it was investigated here whether temperature-induced secondary structure changes of α_S1_-casein were detectable by CD-spectroscopy (Figure 7A). In addition, the melting point of α_S1_-casein was supposed to be determined by nano-DSF (nano-Differential Scanning fluorimetry, Figure S3), as well as a possible temperature dependence of the particle diameter by PCS (Figure 7B). At a temperature of 20° C, α_S1_-casein showed the highest content of α-helical structure (−834,946 Grad·cm^2^·dmol^−1^ at 208 nm) and the largest particle diameter of Ø 637 nm. The weakest molar ellipticity was detected at 30 °C to 40 °C (>720,892 Grad·cm^2^·dmol^−1^ at 208 nm), but α_S1_-casein still exhibited high α-helical content. With temperature increase, the minima were altered from 208 (40 °C) to 205 nm (70 °C) and to 204 nm (100 °C). Such a bathochromic shift of minima indicated the loss of α-helical content, whereby a minimum at 204 nm is characteristic of a high random coil content of a structure. The decrease of α-helical-content was in line with a reduction of particle diameter from Ø 637 nm (20 °C) to Ø 467.6 nm (40 °C), Ø 189.4 nm (70 °C) and Ø 154 nm (90 °C) as determined by PCS. Normally, the ratio of fluorescence at 350/330 nm is expected to be constant in nano-DSF spectra but increases with protein unfolding. The ratio of fluorescence at 350/330 nm decreased for α_S1_-casein from 0.68 (10 °C) to 0.44 (70 °C). This can only be explained by a higher absorbance of larger particles at 350 nm, whereas diameter reduction resulted in lower absorbance at 350 nm, which is in accordance with the results obtained by PCS. Nano-DSF spectra showed a turning point at 80.1 °C for α_S1_-casein. This appears to be caused by a dramatic change in protein structure, e.g., by reaching the melting point [43]. Melting of a protein could result in a loss of molar ellipticity and oligomer diameter. But α_S1_-casein showed high molar ellipticity (−734,946 Grad × cm^2^ × dmol^−1^) and oligomer size (154.9 nm) at 90 °C. Therefore, we attributed the inflection point at 80.1 °C to a change in the conformation of α_S1_-casein. In consequence, α_S1_-casein was considered to be not heat denatureable, but a temperature-induced change of conformation was detectable. As mentioned above, in a previous study, it was reported that α_S1_-casein was no longer binding to TLR4 after heat treatment [8]. As shown here, this non-TLR4-binding α_S1_-casein conformation appears to have a lower α-helical content and a reduced particle diameter in comparison to α_S1_-casein binding to TLR4.

Noteworthy, human α_S1_-casein has higher α-helical content and higher temperature stability compared to bovine α_S1_-casein, which indeed was shown to melt at 65 °C before [17,23]. However, the reduction in oligomer diameter between 20 and 37 °C was similar to that described for bovine α_S1_-casein [44].

In vitro, phosphorylated α_S1_-casein (P-α_S1_-casein) has been shown to be unable to bind to TLR4 [8]. Therefore, the CD-spectra of P-α_S1_-casein at a temperature ranging from 10 to 100 °C (Figure 7C) were compared to those of unphosphorylated α_S1_-casein (Figure 7A). P-α_S1_-casein showed a high α-helical content with a minimum of 206.5 nm at 20 °C (−677,419 Grad·cm^2^·dmol^−1^). The minimum was at a lower wavelength and showed less molar ellipticity compared to α_S1_-casein (−834,946 Grad·cm^2^·dmol^−1^ at 208 nm). This indicated that P-α_S1_-casein had less α-helical content compared to α_S1_-casein.

Incubation of P-α_S1_-casein at 100 °C resulted in a minimum at 203 nm (−826,881 Grad·cm^2^·dmol^−1^). Therefore, incubation resulted in a shift of minimum from 206.5 nm at 20 °C (−677,419 Grad·cm^2^·dmol^−1^) to 203 nm at 100 °C (826,881 Grad·cm^2^·dmol^−1^) at 100 °C for P-α_S1_-casein. P-α_S1_-casein showed an increase of molar ellipticity for the temperature range from 20 to 100 °C, whereas α_S1_-casein showed a loss of molar ellipticity with higher temperatures. This also indicated that P-α_S1_-casein lost α-helical content with incubation at 100 °C and was not predominantly α-helical.

This was supported by in silico structure analyses of α_S1_-casein, showing that phosphorylation sites are in direct neighbourhood to an α-helix (Figure 3B). One known phosphorylation site is located in an α-helix–loop–α-helix motif [11]. Phosphorylations in such a flexible region could lead to a destabilization of neighbouring α-helices due to the additional charge, as suggested by Jakob et al. [45]. Therefore, the structure of P-α_S1_-casein is supposed to have higher flexibility and could result in the exposure of other AA as in α_S1_-casein, which could lead to an altered affinity to itself.

This was investigated by analyzing the binding of P-α_S1_-casein to itself by SPR (Figure 7D). For this purpose, P-α_S1_-casein was immobilized on a CMDP-5 sensor chip, which was monitored by a signal increase of 450 µRIU, and different concentrations of P-α_S1_-casein were injected (0.625 to 5 µM, 5 min, 5 µL/min, RT). By this strategy, a K_D_ value of 0.5 µM was determined for the binding of P-α_S1_-casein to itself. This K_D_ value was four times lower compared to the K_D_ value obtained for the binding of unphosphorylated α_S1_-casein to itself (K_D_ value of 2 µM). P-α_S1_-casein showed higher affinity to itself than α_S1_-casein to itself, P-α_S1_-casein, which does not bind to TLR4 as shown before, had a lower α-helical content, higher random coil content and higher affinity to itself than unphosphorylated α_S1_-casein, binding to TLR-4.

In addition, the influence of the pH value, ranging from pH 2 to pH 10, on the secondary structure of α_S1_-casein (12.5 µM) was investigated by CD-spectroscopy (Figure 7E) to elucidate any pH-dependent structural changes. α_S1_-casein showed α-helical properties at all pH values applied. α_S1_-casein at pH2 showed the typical minima of an α-helical protein at 208 and 222 nm. Furthermore, it showed the strongest molar ellipticity at this pH with −1,345,500 Grad·cm^2^·dmol^−1^ at 208 nm and −966,893 Grad·cm^2^·dmol^−1^ at 222 nm. Therefore, α_S1_-casein should have the highest α-helical at a pH of 2. At alkaline pH values, the minimum was shifted to 205 nm (−1,162,139 Grad·cm^2^·dmol^−1^) at pH 9 (−1,162,139 Grad·cm^2^·dmol^−1^) and to 206 nm (−904,923 Grad·cm^2^·dmol^−1^) at pH 10. Therefore, the lowest α-helical content of α_S1_-casein was detected at alkaline conditions. At pH 5, we detected the less intense minima with a molar ellipticity of −84,040 Grad·cm^2^·dmol^−1^ at a minimum of 208 nm. A decrease in molar ellipticity is associated with stronger interactions between the structural components of a protein and, hence, with a more rigid folding [38]. The isoelectric point of human α_S1_ casein is at pH 5.1, at which it has its highest density.

In order to find out whether these differences in structure observed at different pH values have an influence on the oligomerization of α_S1_-casein, the samples were analyzed by PCS at different pH as well (Figure 7F). The largest oligomers of α_S1_-casein were detected at pH 2 (Ø 826.4 nm). The oligomers of α_S1_-casein at pH 8 (Ø 482.9 nm) were significantly larger compared to oligomers between pH 4 and 7 (Ø 130–73 nm). This is interesting because in the gastrointestinal tract of infants, digestion of milk is supposed to take place under acidic conditions, with a pH range of 3–6 [46]. At a pH of 2, α_S1_-casein formed the largest oligomers and was able to induce an IL-8 secretion via TLR4, as shown in Figure S4. The diameter of oligomers at pH 7 was significantly smaller than that at pH 2. However, α_S1_-casein changed its oligomer diameter dramatically from pH 7 to pH 8, i.e., from 73 (pH 7) to 482.9 nm (pH 8) at 37 °C and to 467.6 nm (pH 7.4) at 40 °C.

In summary, these results show that the α-helical content of αS1-casein was higher, and the oligomer diameter was larger at lower temperatures. Both gained maximum values at a pH of 2. Moreover, TLR4-binding α_S1_-casein was shown to have a higher α-helical content compared to non-TLR4-binding P-α_S1_-casein. The random coil content raised with temperature and phosphorylation. α_S1_-casein did not induce an IL-8 secretion via TLR4 after incubation at 95 °C or after phosphorylation. Therefore, two distinct conformations of α_S1_-casein were proposed: an α-helical TLR4-binding conformation and a less α-helical non-binding conformation. Phosphorylation sites of α_S1_-casein were found in the neighborhood of α-helical regions (Figure 3A). As phosphorylation as well as loss of α-helical content correlated with a loss of TLR4-binding of α_S1_-casein, the TLR4 binding site could be located near the phosphorylation sites within the α-helical regions. The results as obtained indicate that α_S1_-casein induced IL-8 secretion over a wide range of pH and in different oligomeric states. Until today, such pH-resistant activity was only described for immunologically associated proteins of breast milk such as IgG, sIgA and mucin-1, e.g., regulation of proliferation [14,47]. In order to clarify the immunological relevance of these findings, the in vivo phosphorylation state of breast-milk α_S1_-casein and possible ways to its dephosphorylation need to be investigated.

When human α_S1_-casein (1 µM) was incubated for 6 d at RT in a bottomless 96-well plate glued on a glass support, fibrils of 80 nm in length and 20 nm in width were detectable by AFM (atomic force microscopy), and by the incorporation of thioflavin T, a reporter fluorescent dye know to specifically interact with amyloid fibrils. Such fibrils were not formed when P-α_S1_-casein was treated similarly.

### 2.4. α_S1_-Casein Contains a Coiled-Coil Domain

**Figure 8:**
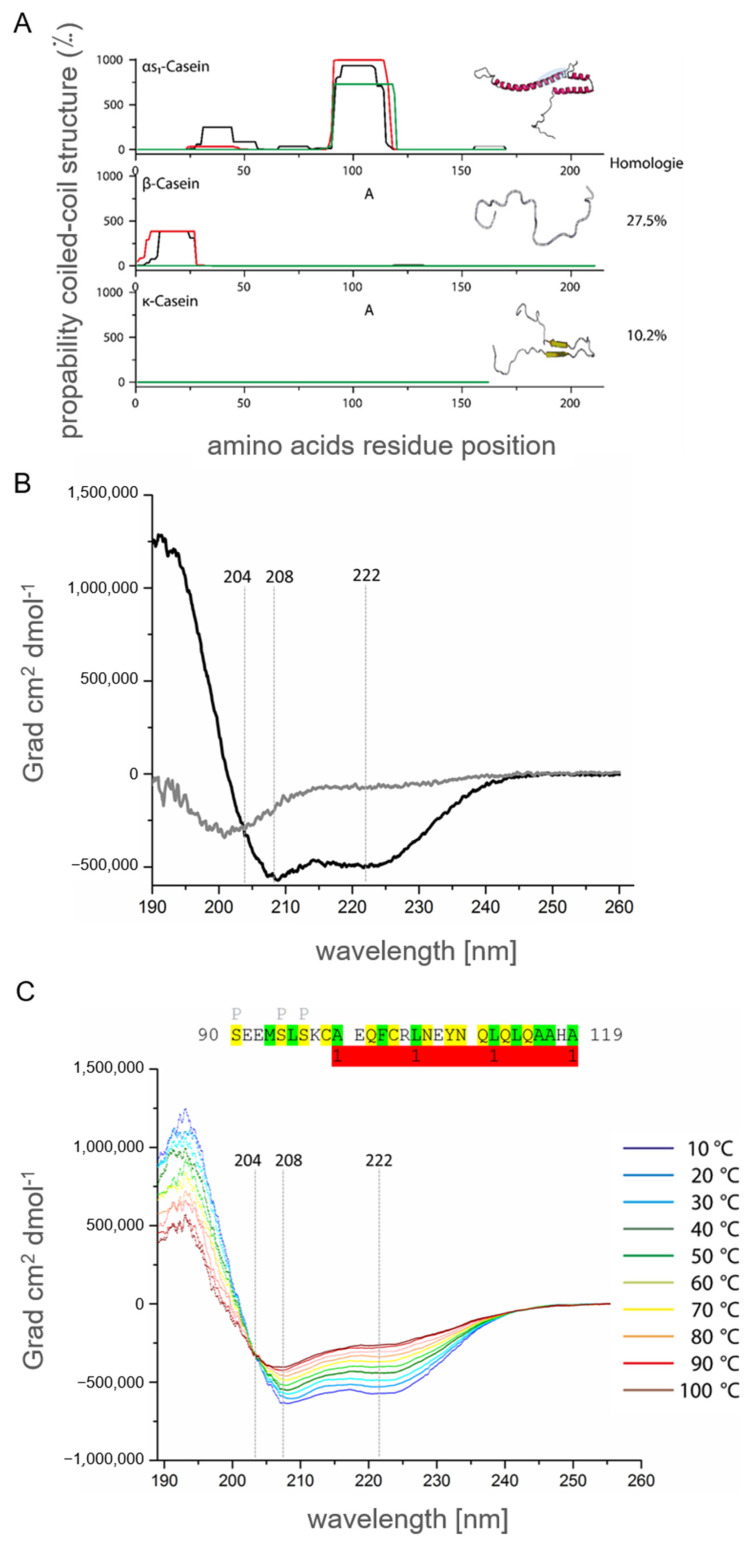
Probability and structural characterization of human α_S1_-casein coiled-coil motive. (**A**) Prediction of coiled-coil motive in AAS of α_S1_-casein, β- and κ-casein with PCOILS (green: window of 14 amino acids; red: window of 21 amino acids; black: window of 26 amino acids) and schematic illustration of secondary structure with RaptorX (pink: α-helix; yellow: β-sheet; white: random coil; blue: predicted coiled-coil motive. Homology of β- and κ-casein to α_S1_-casein was calculated using EMBUSS Needle–Wunsch algorithm (EMBL-EBI, Cambridge, UK). (**B**) CD-spectra of peptide S^91^-A^119^ (5 µM, 2 mm path length) in 30% phosphate buffer/70% trifluorethanol (black) and 100% phosphate buffer (grey). (**C**) CD-spectra of peptide S^91^-A^119^ (^91^SEEMSLSKCA EQFCRLNEYN QLQLQAAHA^119^, 5 µM, 2 mm path length) in 30% phosphate buffer/70% trifluorethanol at different temperatures (10–30 °C blue-light blue; 40–60 °C green-yellow; 70–95 °C orange-dark red). P: potential phosphorylation site; green: hydrophobic amino acids; yellow: polar amino acids; red: coiled-coil domain.

As mentioned in Section 2.1, high molar ellipticity of α_S1_-casein appeared at typical minima of 208 and 222 nm. This phenomenon leads to the investigation of intermolecular interactions of α_S1_-casein, as described in Section 2.2. Furthermore, α_S1_-casein was shown to form oligomers, e.g., tetramers, and was heat stable, as shown in Section 2.3. Interestingly, all these observations are characteristic of a coiled-coil protein [48]. Consequently, we analyzed α_S1_-casein for coiled-coil domains. AAS of α_S1_-casein was analyzed on the prediction of coiled-coil motifs with three different programs. The program COILS (Expasy) compared AAS of α_S1_-casein with AAS of known coiled-coil peptides (Figure 8A). PCOILS (MPI Development Biology, Tübingen) predicted the homology and identity of the AAS of α_S1_-casein to known AAS of coiled-coil motifs, as shown in Figure S5A [49,50,51]. MARCOIL (MPI for Development Biology, Tübingen) used a Hidden Markov Model for the identification of coiled-coil motifs (Figure S5B). In contrast to COILS and PCOILS, MARCOIL was not limited to a certain AAS but considered all amino acids of the protein [50,52]. All three programs predicted a high probability for a coiled-coil motif in the AAS of human α_S1_-casein between AS 102 and 130 (^102^QFCRLNEYN QLQLQAAHAQ EQIRRMNENS^130^). Due to this analysis, α_S1_-casein appears to be the only human casein protein predicted to bind to itself, potentially by a coiled-coil domain (Figure S1). When α_S1_-casein of 17 species was analyzed by the same programs, a high probability for a coiled-coil domain was predicted for hominids (orang utan, gorilla, chimpanzee). The coiled-coil domain within α_S1_-casein of these species was located in the same stretch of the α_S1_-casein AAS (Figure S1). Other regions of these α_S1_-casein AAS did not show a probability for a coiled-coil domain.

For in vitro investigation of this part of the human α_S1_-casein AAS, a peptide corresponding to AA S^91^-A^119^ (^91^SEEMSLSKCA EQFCRLNEYN QLQLQAAHA^119^) of α_S1_-casein, most of the coiled-coil domain was synthesized. Furthermore, the peptide was designed in a way that it would be in direct neighborhood to the phosphorylation site S^89^ of full-length α_S1_-casein. The secondary structure of this peptide was analyzed via CD-spectroscopy (Figure 8B). The spectra showed a minimum below 204 nm, which would be expected for a random coil content. Furthermore, a smaller minimum at 222 nm was detected. The peptide contained parts of a coiled-coil domain and parts of an N-terminal sequence potentially random coil. To isolate the characteristics of the coiled-coil domain, the peptide was analyzed in 70% trifluorethanol (Figure 8C). Trifluorethanol is known to stabilize α-helical content of peptides [53,54]. In trifluorethanol, the secondary structure of the peptide turned out to be merely α-helical with minima at 208 and 222 nm.

For investigating the coiled-coil part of α_S1_-casein by CD spectroscopy, it was assumed that at a certain temperature, the supercoiled α-helix should unfold. The individual α-helices were expected to unfold as well at the same temperature. Thus, two states were expected, a native state and a denatured one, keeping in mind that a coiled-coil structure could also lead to oligomers. A clear indication of a coiled-coil structure is that the corresponding CD spectra at different temperatures show a common point of intersection at ~204 nm up to the complete denaturation of the protein [55]. For this investigation, CD spectra of the peptide S^91^-A^119^ were recorded at temperatures ranging from 10 °C to 95 °C (Figure 8C). Molar ellipticity decreased as temperature increased from −542,048 Grad × cm^2^ × dmol^−1^ at 10 °C to −426,287 Grad × cm^2^ × dmol^−1^ at 90 °C. Moreover, all CD-spectra showed fixed minima at 208 and 222 nm as well as an intersection at 204 nm and −318,760 Grad × cm^2^ × dmol^−1^. The conformation of the peptide was highly stable up to 95 °C. An unfolding of the peptide could not be investigated. Such stability was described for coiled-coil domains before [48]. The intersection at 204 indicated that the peptide could form a coiled-coil structure, which is present in α_S1_-casein as predicted above. This coiled-coil structure is located directly C-terminal to the phosphorylation site SS^89^SSEE of α_S1_-casein and a helix–loop–helix motif as predicted [11]. It, therefore, appears possible that destabilization of the coiled-coil structure by phosphorylation could result in a change of TLR4 agonisticity of α_S1_-casein.

### 2.5. Identification of a TLR4-Stimulating Peptide Derived from α_S1_-Casein

The results as obtained above indicate that the TLR4-binding domain of α_S1_-casein is located in the predicted α-helical region (^16^R–K^98^) containing all known phosphorylation sites, including the most prominent ones S^33^, S^41^, S^71^, and S^89^ [31].

**Figure 9:**
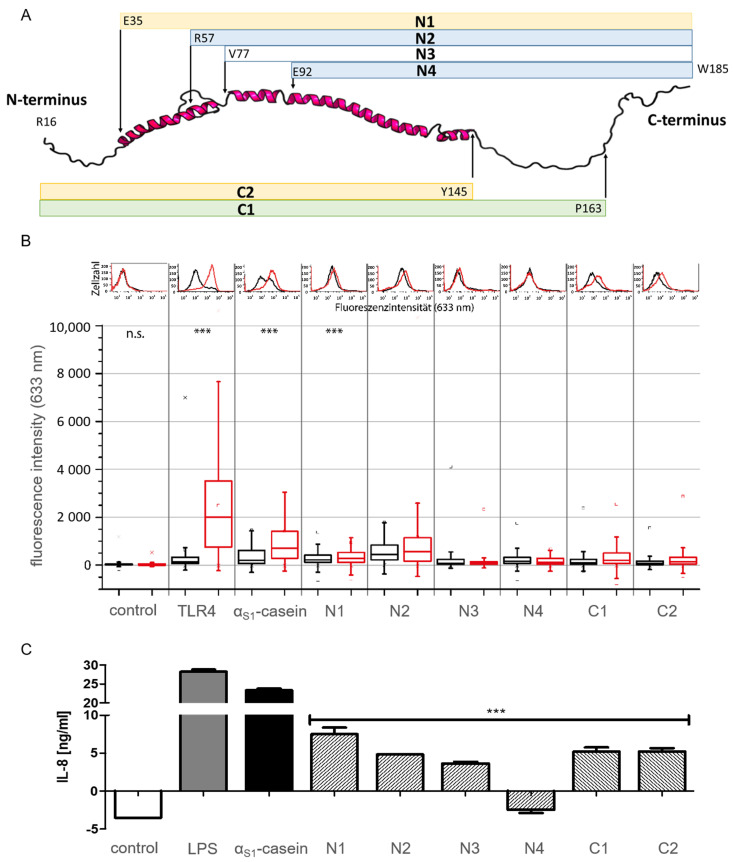
Testing truncated variants of α_S1_-casein for TLR4-binding and induction of IL-8 secretion. (**A**) Schematic illustration of the truncated variants of α_S1_-casein (further information Figure S6; Table S1). (**B**) flow cytometric analysis of N- and C-terminal truncated variants of α_S1_-casein binding to TLR4^+^ (red) and TLR4^−^ (black) cells. Cells were incubated for 24 h with 500 nM of α_S1_-casein variants or without (control). Afterwards, cells were stained with a murine anti-His_6_ IgG (1:100, 1 h, 600 rpm) and caprine Dylight633-anti-murine IgG (1:200, 45 min, 600 rpm). Surface presentation of TLR4 on HEK293 cells (TLR4) was shown by immunostaining the receptor with a murine anti-TLR4 IgG (1:100, 1 h, 600 rpm). (**C**) TLR4^+^ cells were incubated for 24 h with medium (control), LPS, α_S1_-casein or a truncated variant of α_S1_-casein (15 nM). IL-8 was quantified from the supernatants using Sandwich ELISA. An unpaired *t*-test with Welch factor (*** *p* < 0.001; n.s.: not significant) was performed comparing all truncated variants to full-length α_S1_-casein using GraphPad Prism 5 (La Jolla, CA, USA).

To investigate this further, six variants of α_S1_-casein, four truncated at the N-terminus (N1, N2, N3, N4) with an N-terminal His_6_-Tag and two truncated at the C-terminus (C1, C2) with a C-terminal His_6_-Tag. In consequence, these constructs all representing parts of the AAS of α_S1_-casein (Figure 9A and Figure S6, Table S1) were expressed in *E. coli* and purified by NTA column chromatography, analogous to the method described above for the full-size human α_S1_-casein.

The binding of the truncated variants to TLR4 was analyzed by flow cytometry as described before [8]. TLR4^+^ cells (HEK293 cells transfected with TLR4/MD2/CD14) and TLR4^−^ cells (HEK293 cells without TLR4) were incubated with full-length α_S1_-casein and its truncated variants (500 nM), followed by the addition of a murine anti-His_6_ IgG and a caprine Dylight633-antimurine IgG and were finally analyzed by flow cytometry. Cellular fluorescence resulting from the binding of full-size α_S1_-casein and its truncated variants to TLR4^+^ cells is shown in Figure 9B. In addition, IL-8 secretion of TLR4^+^ cells and TLR4^−^ cells after incubation with full-length α_S1_-casein and its truncated variants was analyzed and described before [9], and the results are shown in Figure 9C. Truncated variants N1, N2, C1 and C2 were shown to bind to TLR4^+^ cells, resulting in a 1.2 to 2 times higher fluorescence intensity as obtained after incubation with TLR4^−^ cells. N3 showed only marginal and N4 did not show any difference in fluorescence intensity when incubated with TLR4^+^ cells in comparison to incubation with TLR4^−^ cells (Table S1). Therefore, N3 and N4 appeared to be nonbinders of TLR4. Five of the six truncated variants of α_S1_-caseins induced an IL-8 secretion (N1: 7.5 ng/mL IL-8; N2: 4.8 ng/mL; N3: 3.6 ng/mL; C1: 5.2 ng/mL and C2 5.2 ng/mL). The five truncated variants induced a significantly lower IL-8 secretion compared to full-length α_S1_-casein (23.3 ng/mL IL-8). Truncated variant N4 did not induce any IL-8 secretion.

A clear loss in the induction of IL-8 secretion via TLR4 was shown for α_S1_-casein variants truncated at the N-terminus (N1: 7.5 ng/mL IL-8; N2: 4.8 ng/mL; N3: 3.6 ng/mL; N4: no IL-8 secretion). Hereby, N3 induced an IL-8 secretion of 3.6 ng/mL and showed marginal hints for binding TLR4^+^ cells by flow cytometry as indicated by low fluorescence intensities of 71 for TLR4^+^ and 65 for TLR4^−^ cells. In contrast, N4 did not induce an IL-8 secretion at all (−2.5 ng/mL IL-8). This could be an indication that the AA of N3 missing in the AAS of N4 (V^77^-E^92^) contains a binding motif of α_S1_-casein to TLR4. C-terminal truncated variants C1 and C2 exhibited a similar binding to TLR4 as N1 and N2, as indicated by a 2.3 (C1) to 2.2 times (C2) higher fluorescence intensity when incubated with TLR4^+^ cells in comparison to TLR4^−^ cells. Both induced the secretion of identical amounts of IL-8 (C1, C2: 5.21 ng/mL IL-8) in TLR4^+^ cells. The C-terminal truncations as tested did not lead to a complete loss of binding and induced residual IL-8 secretion, and hence, could be involved in stabilizing the binding region of α_S1_-casein to TLR4.

**Figure 10:**
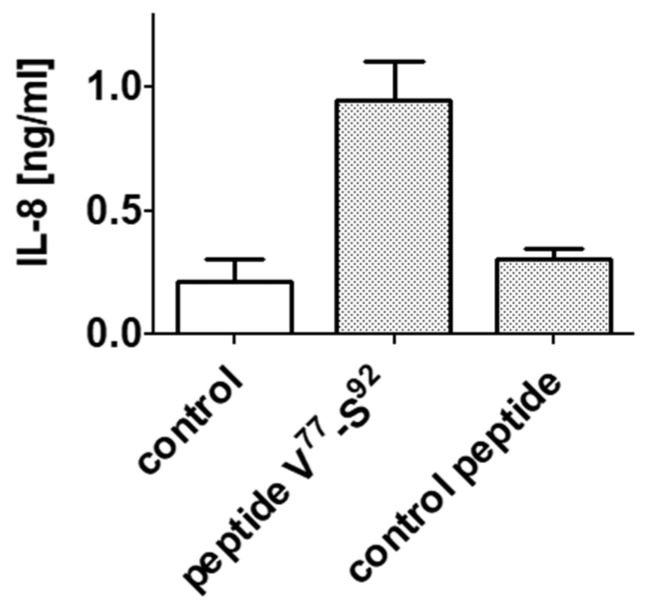
Testing synthetic peptides V^77^-E^92^ and control peptide (each 1.5 µM) for induction of IL-8 secretion. Peptides were incubated with TLR4^+^ cells. Supernatants were analyzed for IL-8 as described above.

Therefore, a peptide corresponding to the AAS of N3 (TLR4 binder) absent in N4 (non-binder) of α_S1_-casein (V^77^-E^92^) was synthesized and tested on induction of IL-8 secretion in TLR4^+^ cells. In addition, a second peptide was synthesized as control and was investigated on IL-8 secretion on TLR4^+^ cells as well. As shown in Figure 10, only peptide V^77^-E^92^ induced an IL-8 secretion (0.95 ng/mL), whereas incubation with the control peptide was not significantly different in IL-8 secretion to the growth medium (0.23 ng/mL IL-8).

## 3. Materials and Methods

### 3.1. Purification of Recombinant α_S1_-Casein, Construction and Isolation of Truncated α_S1_-Casein Variants

Recombinant human α_S1_-casein [8] and P-α_S1_-casein [9] were purified as described before. α_S1_-casein was FITC-labeled as described [9].

The sequence of truncated variants of human α_S1_-casein and rest of the plasmid was amplified from plasmid pET TS001 (coding for CSN1S1 with N-terminal His_6_-Tag) [8] with Phusion DNA polymerase (Thermo Fisher Scientific, Bonn, Germany) using forward (fw) and reverse (rv) oligonucleotides, as listed in Table 1. PCR products were purified according to the manufacturer’s instructions with InnuPREP Plasmid Mini Kit (Analytik Jena, Jena, Germany). The resulting plasmids pET N1, pET N2, pET N3, pET N4, pET C1, pET C2 (Table 1) were transformed into *Escherichia coli* (*E. coli*) strain DH5α (Invitrogen, Carlsbad, CA, USA). Plasmid DNA replication, isolation, transformation into *E. coli* and purification of truncated variants of α_S1_-casein were performed as described before for full-length-α_S1_-casein [8]. Protein purity was evaluated by Coomassie-stained SDS-PAGE. Protein concentration was determined by an indirect ELISA as described [8].

### 3.2. SDS-PAGE Analysis

To evaluate protein purity, samples were diluted 1:1 with strong denaturizing sample buffer (100 µM Tris/HCl (pH 6.8), 4% SDS, 200 mM DTT, 0.2% bromophenol blue and 20% glycerol) boiled for 20 min at 95 °C. For analyzing oligomers, samples were diluted 1:1 once in weak denaturizing sample buffer (only 0.2% SDS, without DTT) in sample buffer (without DTT) and then incubated for 20 min at RT. Further samples were diluted in sample buffer with 0, 10, 100 mM DTT and boiled at 95 °C for 20 min.

All samples were loaded onto an SDS-Gel containing 15% acrylamide with PAGE-ruler prestained protein marker (Fermentas, St. Leon-Roth, Germany) as molecular weight standard. After separation (80 V protein focus, 120 V protein separation), proteins were stained with Coomassie brilliant blue G250 (Serva, Heidelberg, Germany) for protein purity analysis and with silver staining for analyzing oligomers. For silver-staining, SDS-PAGE gel was fixed for 1 h (50% ethanol, 5% acidic acid). Gel was washed with 50% ethanol (10 min), twice with water (10 min), with sodium thiosulfate (0.02%) and twice with water (5 min). Gel was incubated for 30 min in 0.1% silver nitrate solution, quickly washed with water and developed in a solution of 0.04% formaldehyde and 2% sodium carbonate. The reaction was stopped in 5% acidic acid.

### 3.3. Secondary Structure Analysis by CD- and ATR-FTIR-Spectroscopy

All samples were transferred into a 10 mM NaH_2_PO_4_/Na_2_HPO_4_ buffer (pH 7.2, chloride-free buffer) for CD measurements. CD spectra were conducted on a Jasco J-815 spectrometer with a Jasco PTC-348WI Peltier-type temperature control system (Jasco Corp, Hachioji, Japan) at constant nitrogen flow. Far-UV CD spectra were measured with 2 mm path length quartz cuvette. Human α_S1_-casein was recorded at a concentration of 12.5 µM. Peptides were analyzed with a concentration of 5 µM in 30% phosphate buffer/70% trifluorethanol. Spectra were recorded from 190 to 260 nm with a resolution of 0.1 nm (100 nm/min). The final spectra were corrected by subtracting the corresponding baseline spectrum and secondary analysis was estimated [38].

Next, 200 µL α_S1_-casein (1 mg/mL in PBS with 4 M Urea) were dried (60 °C) and suspended in 50 µL water. IR spectra were measured from 1300 to 1700 cm^−1^ on an ATR-FTIR MIRacle 10 (Shimadzu, Kyoto, Japan) with a resolution of 2.0 cm^−1^/mean of 100 interferogramms. Vibrations of samples were analyzed for characteristics of secondary structure [56]. Spectra analysis was performed by Origin Lab (Northampton, MA, USA).

### 3.4. Microscale Thermophoresis Assay (MST)

For the determination of K_D_ values, Microscale Thermophoresis (Monolith NT.115 (NanoTemper Technologies GmbH, München, Germany) was used [57]. Removal of fluorophore excess was controlled by comparison of different concentrations of labeled FITC-α_S1_-casein. For thermophoresis experiments, 10 µL of FITC-α_S1_-casein was mixed with 10 µL of α_S1_-casein in serial dilution (50 µM diluted 1:1 with buffer to 0.031 µM), sonicated for 15 min assuming monomerization and incubated for 1 h at 37 °C assuming renaturation as described for human milk micelles [58]. Subsequently, samples were analyzed by MST (LED: 40–60%; MST: 50%, 30 s). Data were analyzed by Graphpad Prism 5.0. All measurements were performed in a 20 mM HEPES buffer (pH 7.2) with 0.5% BSA and 0.0025% Tween-20. Thermophoresis data analysis was performed in three independent experiments.

### 3.5. Surface Plasmon Resonance Spectroscopy Assay (SPR)

The binding of α_S1_-casein to itself was monitored using an SPR Device 8700 system (Reichert Life Sciences, Depew, NY, USA) based on the protocol described before for bovine α_S1_-casein [21]. An SPR sensor gold-carboxymethyldextran chip (CMDP-5, Xantec Bioanalytics, Düsseldorf, Germany) was used for protein immobilization and kinetic detection. This chip was equilibrated with water (30 min) and activated with N-hydroxysuccimid (100 mM, 10 min) and 1-ethyl-3-(3-dimethylaminopropyl)carbodiimide hydrochloride (100 mM, 10 min), both in 50 mM 2-(N-morpholino)-ethansolfonacid (pH 5). α_S1_-casein or P-α_S1_-casein in 10 mM sodium acetate (pH 4.1) were immobilized until a signal increase of 450 µRIU. Free reaction sites were quenched by ethanolamine (1 M, pH 8.5).

Different concentrations of α_S1_-casein or P-α_S1_-casein (from 0.625 to 5 µM in 10 mM HEPES, 150 mM NaCl, pH 7.2) were injected (5 min, 5 µL/min). Afterwards, dissociation was monitored by injecting running buffer for 10 min (5 µL/min). The binding partner was removed by injection of 10 mM NaOH (5 min) and washing. Chip was regenerated by three injections of 4 M urea (2 min, 5 µL/min) and incubation (37° C, 30 min).

### 3.6. Photon Correlation Spectroscopy

α_S1_-casein (1 mL, 50 µM) in phosphate buffer was incubated for 3 h at 37 °C. Samples were transferred into a quartz cuvette. Proteinoligomer diameter and oligomers’ population PI were analyzed by PCS (Zetasizer NanoZS3600, Malvern Instruments, Worcestershire, UK). Before each measurement, samples were equilibrated for at least 2 min. The mean of 15 single measurements was recorded. Samples were recorded with an attenuator value of 8. Temperature denaturation was recorded by a heating rate resolution of 0.5 °C.

### 3.7. Cell Culture, Stimulation and Flow-Cytometric Binding Experiments

For identification of a binding domain of α_S1_-casein to TLR4, TLR4^+^ cells (HEK293 cells transfected with TLR4/MD2/CD14) and TLR4^−^ cells were cultured according to manufacturer’s protocols (InvivoGen, San Diego, CA, USA).

For flow cytometry binding assay, TLR4^+^ and TLR4^−^ cells (500,000 cells in 500 µL medium, 48-well plate) were seeded out and incubated for 24 h (37 °C, 5% CO_2_). The medium was refreshed with the addition of α_S1_-casein or a truncated variant of α_S1_-casein (500 nM). After 24 h of incubation, cells were washed with 500 µL PBS, incubated in 500 µL PBS (10 min, 37 °C, 5% CO_2_), detached and transferred into a 1.5 mL Eppendorf reaction tube. Cells were centrifuged (500× *g*, 5 min, RT) and incubated with murine primary antibody, either anti-His_6_ IgG for detection of α_S1_-casein and truncated variants or anti-TLR4 for control of TLR4-expression (1 h, 1:100, RT, 600 rpm). Cells were washed three times with 500 µL PBS and incubated with secondary caprine Dylight633 antimurine IgG (45 min, 1:200, RT, 600 rpm). Afterwards, cells were washed and fixed with 1% paraformaldehyde (100 µL, 10 min, RT, 600 rpm). Cells were washed, suspended in PBS, filtered (30 µm filter, CellTrics Sysmex, Hamburg, Germany) and analyzed by flow cytometry (BD FACSaria^TM^ III, Becton Dickinson, Heidelberg, Germany) with extinction: 633 nm/emission: 660/20 nm (500 V).

For stimulation experiments, TLR4^+^ and TLR4^−^ cells (50,000 cells in 200 µL medium) were seeded out in a 96-well microtiter plate and cultivated for 24 h (37 °C, 5% CO_2_). Afterwards, the medium was refreshed with the addition of α_S1_-casein, a truncated variant of α_S1_-casein (15 nM) or one synthetic peptide (V^77^-E^92^ and V^77^-A^119^, each 1.5 µM, obtained from GL Biochem Ltd., Shanghai, China). IL-8 concentration of the supernatant was determined after 24 h incubation using a Human CXCL8/IL-8 DuoSet ELISA Kit and Human TNF-α DuoSet ELISA Kit (R & D Systems, Minneapolis, MN, USA) following the manufacturer’s instructions with the modifications described before by Saenger et al. [9].

### 3.8. Studies on Human α_S1_-Casein Fibrillation (Thioflavin T and Atomic Force Microscopy)

The formation of fibrils of human α_S1_-casein with 10 µM Thioflavin T (ThT) was monitored in an Infinite M200 Pro plate reader (TecanGroup, Männerdorf, Switzerland). ThT (1 mg/mL in PBS) was sterilized using a 0.22 µM filter (Diagonal, Germany). The final concentration was determined by measuring its absorbance at 412 nm using a molar extinction coefficient of 36,000/M·cm [59]. Different concentrations of α_S1_-casein (0, 6.25, 12.5 and 25 µM) were incubated with ThT (10 µM, using a total volume of 200 µL, 96-well plate (Greiner BioOne, Frickenhausen, Germany)). The microtiter plate was sealed to prevent evaporation and incubated for 60 h at 37 °C. Fluorescence was measured every hour (excitation: 440 nm/emission: 485 nm).

For AFM sample preparation, human α_S1_-casein (25 µM) was incubated for 6 d at 37 °C in 1.5 mL Eppendorf reaction tube. Samples were centrifuged (20 min, 20,000× *g*, 4 °C) and suspended in 10 µL ddH_2_O. The suspension was transferred onto a freshly cleaved mica chip (NanoAndMore, Wetzlar, Germany). This chip coated with α_S1_-casein was washed five times with 100 µL ddH_2_O and dried under a constant flow of nitrogen gas for 20 min. A Bruker Dimension 3100 atomic force microscope, equipped with a Nanoscope IIIa controller (Bruker, Karlsruhe, Germany) was used. Measurements were performed in tapping mode with n-type silicon cantilevers (HQ:NSC14/Al BS, nominal tip radius <10 nm, typical resonant frequency of about 160 kHz and a nominal spring constant of 5 N/m; manufactured by µmash, Sofia, Bulgaria). Nanoscope analysis software version 1.5 was used for data analysis.

## 4. Conclusions

Secondary structure analysis revealed that TLR4-agonistic α_S1_-casein was mostly α-helical but also able to adopt a partial β-sheet structure. The increased α-helical content was associated with the binding of α_S1_-casein to TLR4 and IL-8 secretion. Whereas the α-helical structure was stable over a wide range of pH and up to 80 °C (as well as IL-8 secretion), it was substantially altered by phosphorylation, which omitted TLR4-binding and IL-8 secretion.

Within α_S1_-casein a TLR4-agonistic peptide (V^77^-E^92^) was identified. Moreover, a coiled-coil domain was demonstrated at the same position of α_S1_-casein from primates, such as human, orang-utan, gorilla or chimpanzee, but not from other mammals. Phosphorylation of α_S1_-casein led to higher flexibility, higher affinity to itself (K_D_ value: 0.5 µM, non-phosphorylated K_D_ value: 2 µM), formation of random aggregates and loss in structural constraints in comparison to α_S1_-casein.

The differences in conformation regulated by phosphorylation appear to be a kind of switch between two different states of α_S1_-casein, which could be related to two different functions. On the one hand, a nutritional role of breast milk α_S1_-casein and, on the other hand, an immunostimulatory role of α_S1_-casein by binding TLR4, inducing proinflammatory processes and immune cell maturation, as has been shown before.

In a previous study, we were able to show that breast milk from breastfeeding mothers contained phosphorylated α_S1_-casein and identified the phosphorylation sites by a targeted MS approach. In a further study, we could show that having been breastfed leads to a lifetime IgG response against unphosphorylated α_S1_-casein. As synopsis with the data of the present study, it can be hypothesized that phosphorylated α_S1_-casein in breast milk is dephosphorylated during or after breastfeeding and enters the intestine of the suckling. Not at least due to its pH stability, it is resorbed—in an unphosphorylated conformation—and can fulfill its immunostimulatory function. It may serve as a signal for the infant that he is out of the womb and, from now on, needs to take care on his own for his immune status. Systematic and continuous analyses of the α_S1_-casein content in breast milk of breastfeeding mothers as well as a timely resolution following the onset of the suckling’s immune system, would be the next steps to support this hypothesis, investigating formula-fed infants as control. The comparison of key immune parameters in breastfed and formula-fed persons of different ages could provide further elucidating insights.

## Figures and Tables

**Table 1:** List of α_S1_-casein truncated variants, plasmids coding for these truncated variants and used oligonucleotides for construction of these plasmids.

Resulting Protein	Corresponding to AAS of α_S1_-Casein	Plasmid	Oligonucleotide
N1	35–185	pET N1	Fw: CACCATCACCATCATGAGCCTATACCATTAGAATCAAGAGAGGAA Rv: TAGCASGCCGGATCCGTTACCACTGTAGCATGACGTTATTTTTTTCATA
N2	57–185	pET N2	Fw: CACCATCACCATCATAGAGAAAAACAGACTGATGAAATCAAGGATACTAGG: Rv: TAGCASGCCGGATCCGTTACCACTGTAGCATGACGTTATTTTTTTCATA
N3	77–185	pET N3	Fw: CACCATCACCATCATGAAATGTCTCTCAGTAAGTGTGCGGAACAG Rv: TAGCASGCCGGATCCGTTACCACTGTAGCATGACGTTATTTTTTTCATA
N4	93–185	pET N4	Fw: CACCATCACCATCATTTGTGGCAGAGCCTGAGAAG Rv: TAGCASGCCGGATCCGTTACCACTGTAGCATGACGTTATTTTTTTCATA
C1	16–163	pET C1	Fw: GTGGTGGTGCTCGAGCGGTGGGAAAGGAACATAC Rv: CTCGAGCACCACCACC
C2	16–145	pET C2	Fw: GTGGTGCTCGAGGTAGGCAGCAAGTTGGTT Rv: CTCGAGCACCACCACC

## Data Availability

Data is contained within the article and Supplementary Material.

## Supplementary Materials

The following supporting information can be downloaded at: https://www.mdpi.com/article/10.3390/ijms25031743/s1.

## Abbreviations

AAS, amino acid sequence; AFM, atomic force microscopy; ATR-FTIR, Attenuated Total Reflection-Fourier Transform Infra-Red; CD, circular dichroism; CK2, human protein kinase CK2; FITC, fluorescein isothiocyanate; IDP, intrinsically disordered protein; IgG, Immunoglobulin G; IL-8, Interleukin 8; P-α_S1_-casein, phosphorylated α_S1_-casein; MST, microscale thermophoresis; nano-DSF, nano-Differential Scanning Fluorometry; PCS, photon correlation spectroscopy; PI, polydispersion index; SPR, surface plasmon resonance spectroscopy; ThT, thioflavin T; TLR4, Toll-like receptor 4; TLR4^+^ cells, HEK293 cells transfected with TLR4/MD2/CD14; TLR4^−^ cells, HEK293 cells without TLR4/MD2/CD14.
