# Supplementary material for: Structural Analysis of Breast-Milk αS1-Casein: An α-Helical Conformation Is Required for TLR4-Stimulation
Source: Int J Mol Sci. 2024 Feb 1;25(3):1743. doi: 10.3390/ijms25031743 (PMC10855866; doi:10.3390/ijms25031743)
Supplement: Supplementary file 1 [file ijms-25-01743-s001.zip › ijms-2762484-supplementary.pdf]

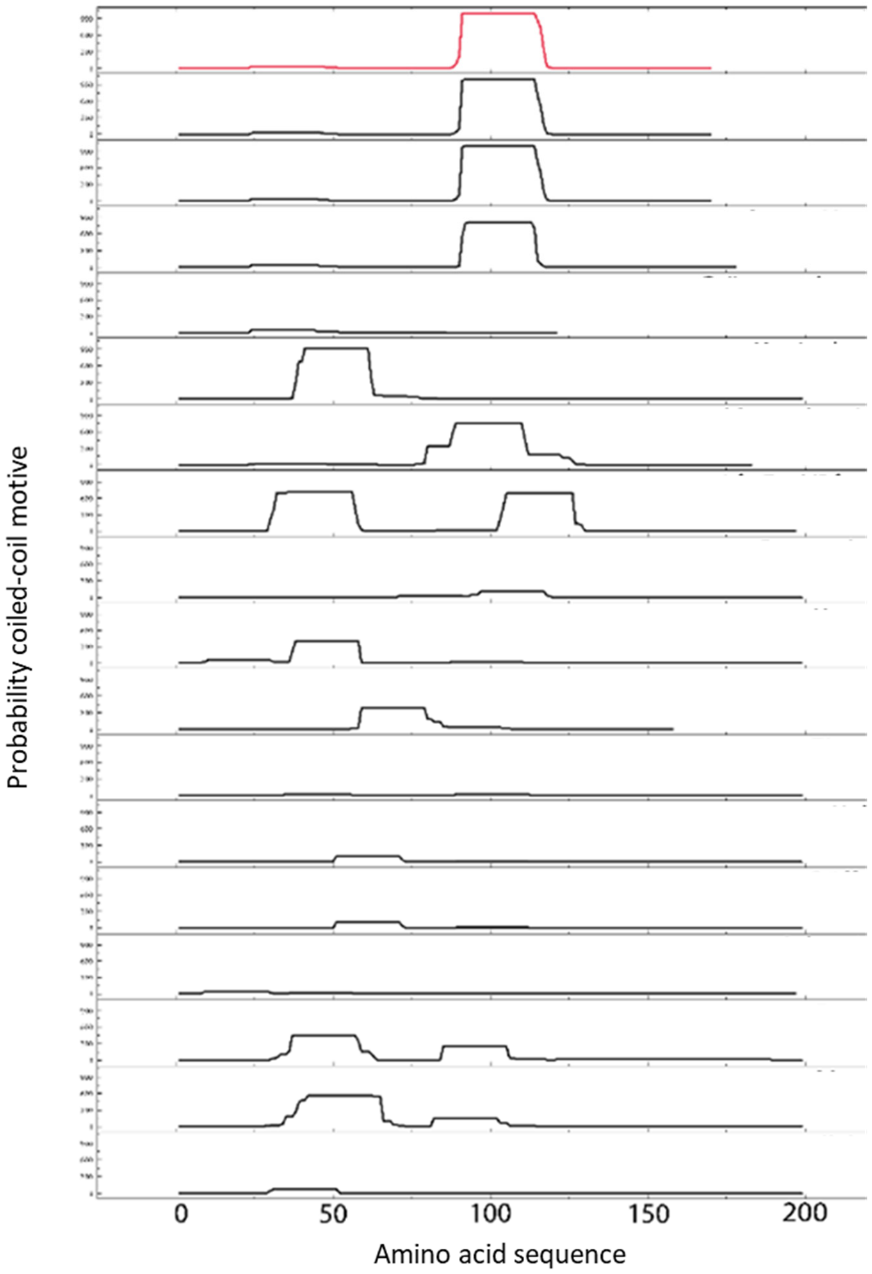


| **Species** | **Identity (%)** | **Homology (%)** | **Amino acids** | **Uniprot** |
|---|---|---|---|---|
| Homo sapiens (Human) | - | - | 170 | P47710 |
| *Pan troglodytes (Chimpanzee)* | 97 | 99 | 170 | H2QPK8 |
| Gorilla *(Western lowland gorilla)* | 95 | 98 | 154 | G3RGP3 |
| *Pongo abelii (Sumatran orangutan)* | 92 | 95 | 170 | H2PDG9 |
| *Chlorocebus sabaeus (Green monkey)* | 82 | 89 | 171 | A0A0D9QY08 |
| Oryctolagus cuniculus (Rabbit) | 42 | 56 | 200 | P09115 |
| Cavia porcellus (Guinea pig) | 39 | 55 | 183 | P04656 |
| *Equus asinus (Donkey)* | 39 | 52 | 202 | P86272 |
| *Camelus dromedarius (Dromedary)* | 39 | 50 | 215 | O97943 |
| *Felis catus (Cat)* | 35 | 48 | 155 | A0A337S0Z8 |
| *Canis lupus familiaris (Dog)* | 32 | 47 | 143 | J9P7W6 |
| Capra hircus (Goat) | 34 | 46 | 199 | P18626 |
| *Bos taurus (Bovine)* | 33 | 45 | 199 | P02662 |
| *Bubalus bubalis (Domestic water buffalo)* | 33 | 43 | 199 | O62823 |
| Sus scrofa (Pig) | 35 | 43 | 191 | P39035 |
| Rattus norvegicus (Rat) | 29 | 38 | 269 | P02661 |
| Mus musculus (Mouse) | 27 | 36 | 298 | P19228 |
| *Macropus eugenii (Tammar wallaby)* | 18 | 31 | 222 | P28549 |

**Supplementary Figure 1: Comparison of the AAS of human $\alpha_{S1}$-casein with $\alpha_{S1}$-casein from other species.** Probability for coiled coil structures (PCOILS) of 18 mammalian $\alpha_{S1}$-caseins AAS (left). Identity and homology of human $\alpha_{S1}$-casein to 17 other species $\alpha_{S1}$-casein was calculated by EMBOSS Needle-Wunsch (EMBL-EBI, Cambridge, UK) (right).

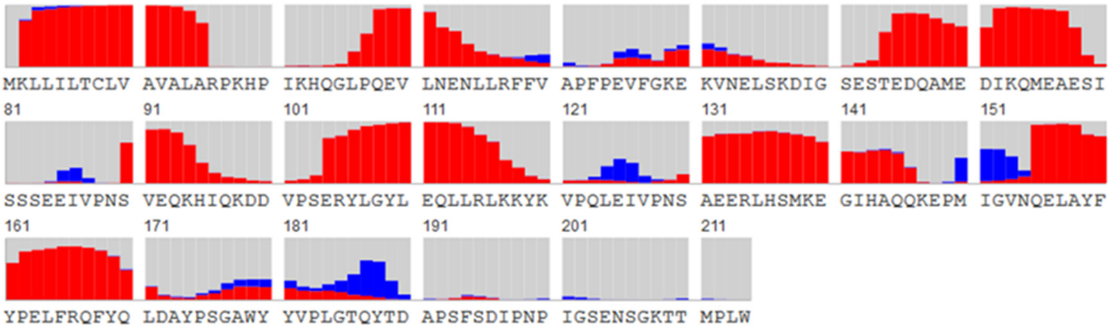


**Supplementary Figure 2:** Secondary structure prediction of the AAS of bovine $\alpha_{S1}$-casein with signal peptide (*grey*: random coil structure; *red*: α-helix; *blue*: β-sheet).

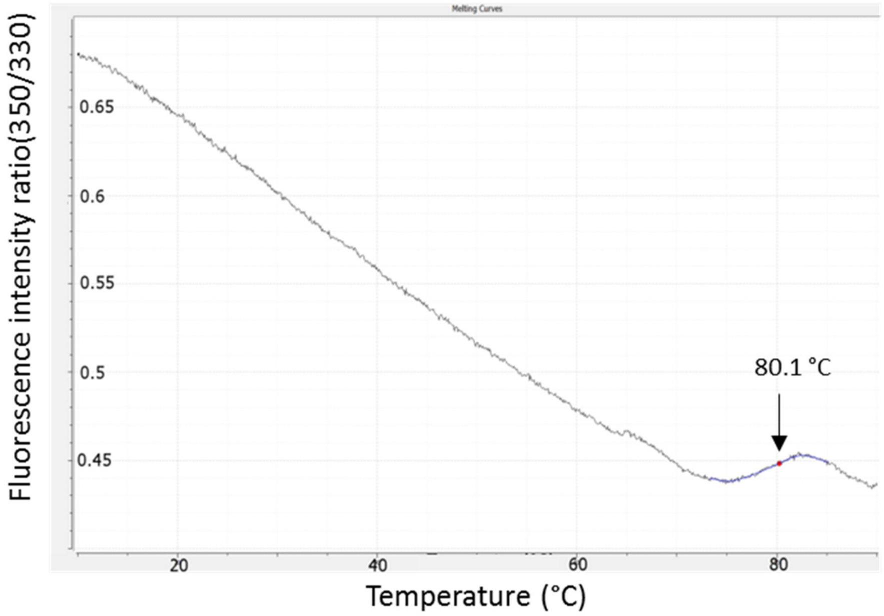


**Supplementary Figure 3: Melting point of human $\alpha_{S1}$-casein determined by nano-Differential Scanning Fluorometry.**

Nano-Differential-Scanning Fluorimetry (Prometheus Series, Nanotemper Technologies, Munich, Germany) was used to determine protein stability [43]. Fluorescence of $\alpha_{S1}$-casein (50 µM) was detected at 350 nm and 330 nm. Sample was heated from 10 °C to 90 °C and monitored. Ratio of the fluorescence at 350 nm to 330 nm was recorded.

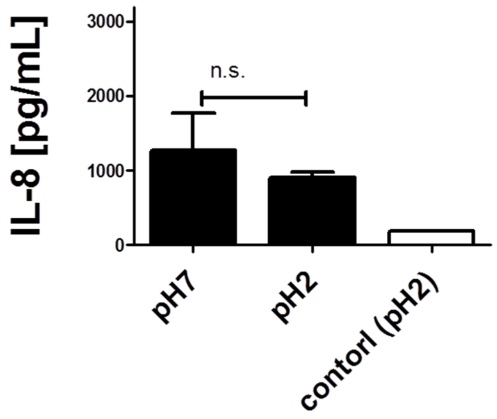


**Supplementary Figure 4: Effect of incubation at pH2 on $\alpha_{S1}$-casein-induced IL-8 secretion.**
$\alpha_{S1}$-casein was incubated at pH 2 and tested for induction of an IL-8 secretion *via* TLR4 as described by Saenger et al. (2019)[9]. Resulting IL-8 secretion of acidic treated $\alpha_{S1}$-casein (pH 2) was not significant different from $\alpha_{S1}$-casein incubated at pH 7.4. As a control, TLR4+ cells were incubated with a buffer at pH2 (without the addition of $\alpha_{S1}$-casein).

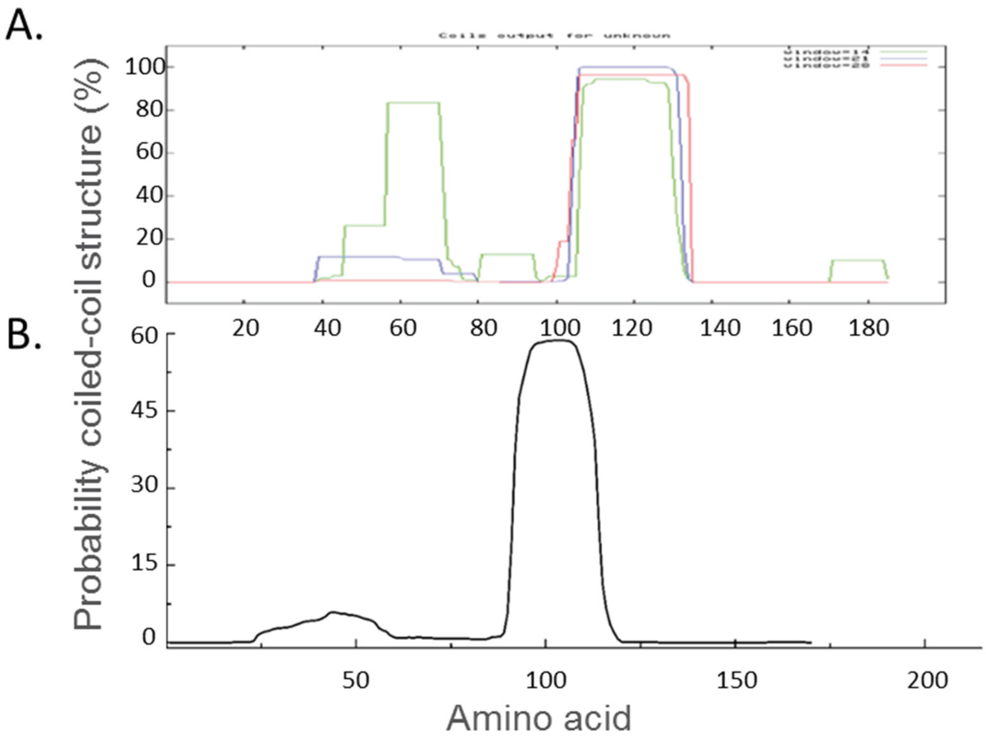


**Supplementary Figure 5: Probability of coiled-coil formation in AAS of human $\alpha_{S1}$-casein.**

**A**. Prediction of a coiled-coil in the AAS of $\alpha_{S1}$-casein using COILS (green: window of 14; blue: window of 21; red: window of 26). **B.** Prediction of a coiled-coil in the AAS of $\alpha_{S1}$-casein using MARCOILS.

A

```
           10         20         30         40         50
αS1-Casein        RPKLP LRYPERLQNP SESSEPIPLE SREEYMNGMN
N1(35-185)                                EPIPLE SREEYMNGMN
N2(57-185)
N3(77-185)
N4(93-185)
C1(16-163)        RPKLP LRYPERLQNP SESSEPIPLE SREEYMNGMN
C2(16-145)        RPKLP LRYPERLQNP SESSEPIPLE SREEYMNGMN

        60         70         80         90        100
RQRNILREKQ TDEIKDTRNE STQNCVVAEP EKMESSISSS SEEMSLSKCA
RQRNILREKQ TDEIKDTRNE STQNCVVAEP EKMESSISSS SEEMSLSKCA
      REKQ TDEIKDTRNE STQNCVVAEP EKMESSISSS SEEMSLSKCA
                            VAEP EKMESSISSS SEEMSLSKCA
                                             EEMSLSKCA
RQRNILREKQ TDEIKDTRNE STQNCVVAEP EKMESSISSS SEEMSLSKCA
RQRNILREKQ TDEIKDTRNE STQNCVVAEP EKMESSISSS SEEMSLSKCA

       110        120        130        140        150
EQFCRLNEYN QLQLQAAHAQ EQIRRMNENS HVQVPFQQLN QLAAYPYAVW
EQFCRLNEYN QLQLQAAHAQ EQIRRMNENS HVQVPFQQLN QLAAYPYAVW
EQFCRLNEYN QLQLQAAHAQ EQIRRMNENS HVQVPFQQLN QLAAYPYAVW
EQFCRLNEYN QLQLQAAHAQ EQIRRMNENS HVQVPFQQLN QLAAYPYAVW
EQFCRLNEYN QLQLQAAHAQ EQIRRMNENS HVQVPFQQLN QLAAYPYAVW
EQFCRLNEYN QLQLQAAHAQ EQIRRMNENS HVQVPFQQLN QLAAY
EQFCRLNEYN QLQLQAAHAQ EQIRRMNENS HVQVPFQQLN QLAAYPYAVW

       160        170        180
YYPQIMQYVP FPPFSDISNP TAHENYEKNN VMLQW
YYPQIMQYVP FPPFSDISNP TAHENYEKNN VMLQW
YYPQIMQYVP FPPFSDISNP TAHENYEKNN VMLQW
YYPQIMQYVP FPPFSDISNP TAHENYEKNN VMLQW
YYPQIMQYVP FPPFSDISNP TAHENYEKNN VMLQW

YYPQIMQYVP FPP
```

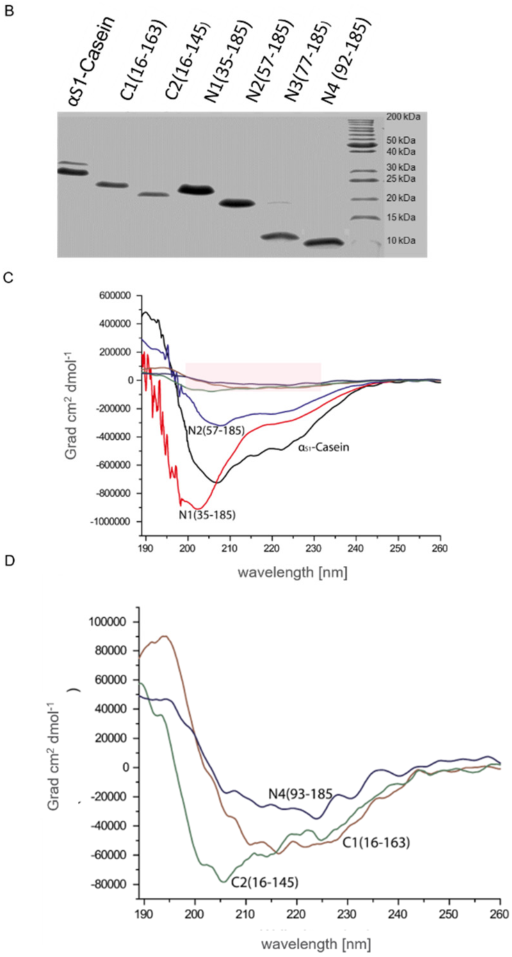


**Supplementary Figure 6: Characterization of truncated variants of human $\alpha_{S1}$-casein.**

**A.** AAS of truncated variants of human $\alpha_{S1}$-casein. **B.** Coomassie stained SDS-PAGE gel (15%) of denaturized truncated variants of $\alpha_{S1}$-casein (2% SDS, 100 mM DTT, 95 °C, 20 min). Compare bands to molecular mass of truncated variants (**suppl. Table 1**). **C, D.** CD-spectra of truncated variants of $\alpha_{S1}$-casein (12.5 µM) in 10 mM phosphate buffer.

**Supplementary Table 1: Selected features of the investigated truncated variants of human $\alpha_{S1}$-casein.**

| | Amino acids | Molecular mass (kDa) | $TLR4^+$ cells (median fluorescence intensity) | $TLR4^-$ cells (median fluorescence intensity) | Fluorescence intensity ($TLR4^+/TLR4^-$) | Induced IL-8 secretion (ng/ml) |
|---|---|---|---|---|---|---|
| $\alpha_{S1}$-casein | R16-W185 | 21 | 703 | 193 | 3.6 | 23.3 |
| N1 | E35-W185 | 17,8 | 272 | 221 | 1.2 | 7.5 |
| N2 | R57-W185 | 15,2 | 564 | 432 | 1.3 | 4.8 |
| N3 | V77-W185 | 12,8 | 71 | 65 | 1.1 | 3.6 |
| N4 | E93-W185 | 11,2 | 121 | 154 | 0.8 | -2.5 |
| C1 | R16-P163 | 17,5 | 201 | 87 | 2.3 | 5.2 |
| C2 | R16-Y145 | 15,2 | 134 | 60 | 2.2 | 5.2 |
